# Supplementary material for: The evolution of patterning during movement in a large-scale citizen science game
Source: Proc Biol Sci. 2021 Jan 13;288(1942):20202823. doi: 10.1098/rspb.2020.2823 (PMC7892415; doi:10.1098/rspb.2020.2823)
Supplement: Supplementary Material [file rspb20202823supp1.docx]

**The evolution of patterning during movement in a large-scale citizen science game**

Anna E. Hughes, David Griffiths, Jolyon Troscianko, Laura A. Kelley

**Supplementary material**

**S1. Pattern generation process**

Targets were generated in a hierarchical manner, as shown in Figure S1. The ‘tree structure’ of the program determining the target pattering is composed of two different types of node. One type of node is the ‘terminal node’ that is found on the outer ring (‘layer’) of the tree. There were two possible variants of terminal node (each chosen with a probability of 50%). One variant was a flat image of a specific RGB colour (always greyscale) and alpha (transparency) value. The second variant of terminal node consisted of a specific pre-generated image; there were 66 different initial images from a range of different categories, including striped patterns, spotted patterns and noise patterns, and with a range of spatial scales (see Figure S2 and online repository of base images at https://github.com/fo-am/dazzlebug/tree/master/client/htdocs/images/themes/huge). These base images could also be moved using an x-offset and a y-offset value (with the patterns wrapping around the target) and rotated (in radians). The other type of node was the ‘combination node’. Here, two image inputs were combined to generate a new image using one of the following randomly selected combination operations:

- Source-over: the second image was drawn on top of the first image
- Source-atop: the second image was drawn only when it overlapped the first image (i.e. the second image was not drawn on the transparent parts of the first image)
- Destination-over: the second image was drawn behind the first image
- Lighter: when both images overlapped, the new colour was determined by adding the colour values
- Xor: the new image was made transparent when both the first and second images overlapped, and was drawn normally everywhere else


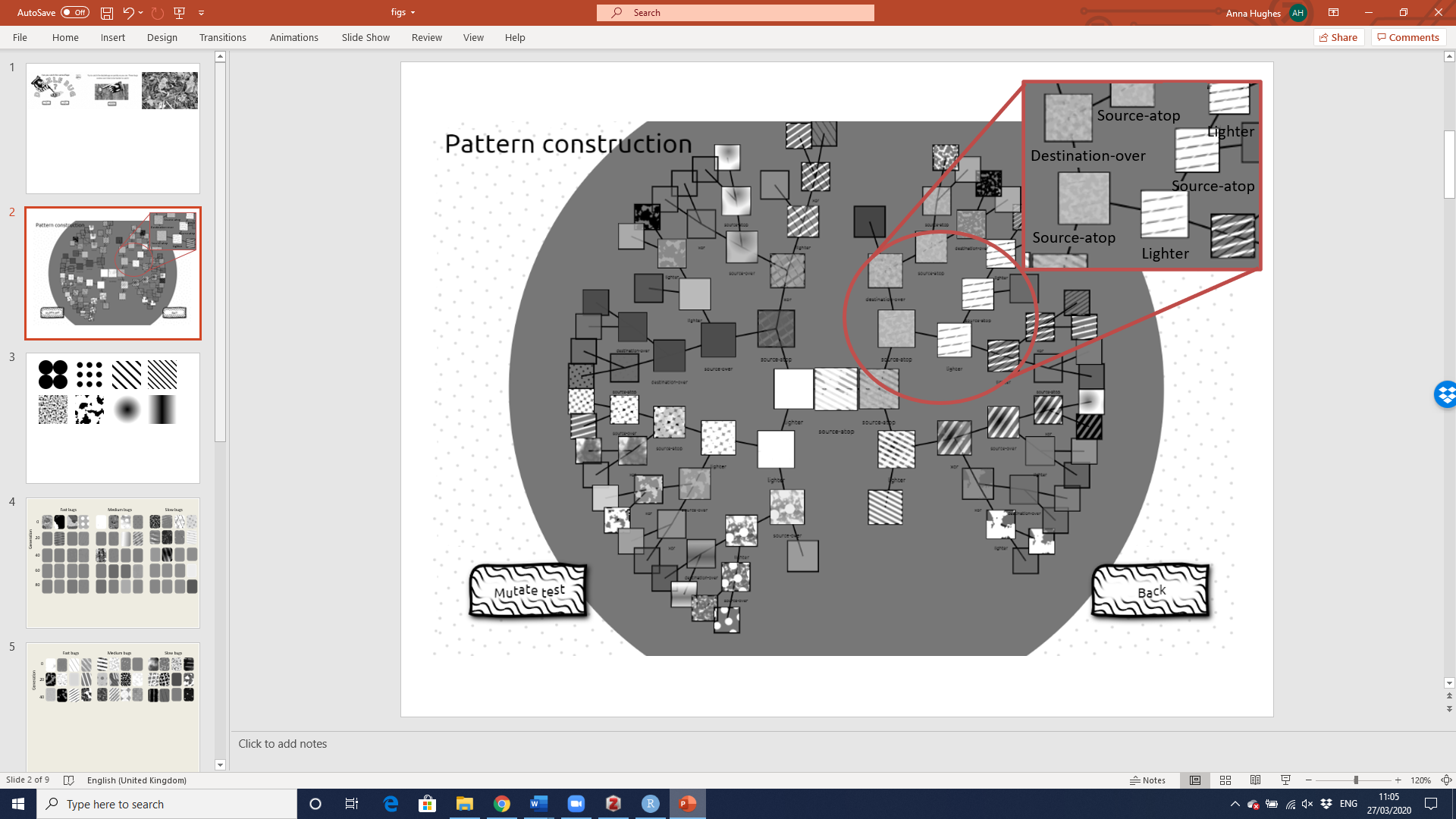


Figure S1: Schematic to show how targets are generated (similar examples are available to view on the online version of the game at <http://www.dazzle-bug.co.uk/>). Each target (shown as the central image in this diagram) can be thought of as being the top point of a ‘tree’ made up from a range of different images, combined in different ways. The ‘nodes’ of the tree are combination operations, that each take two images as input. The tree continues until the outer edge of the circle, where the terminal nodes are made up of the base images. The magnified region shows the combination operations more clearly.


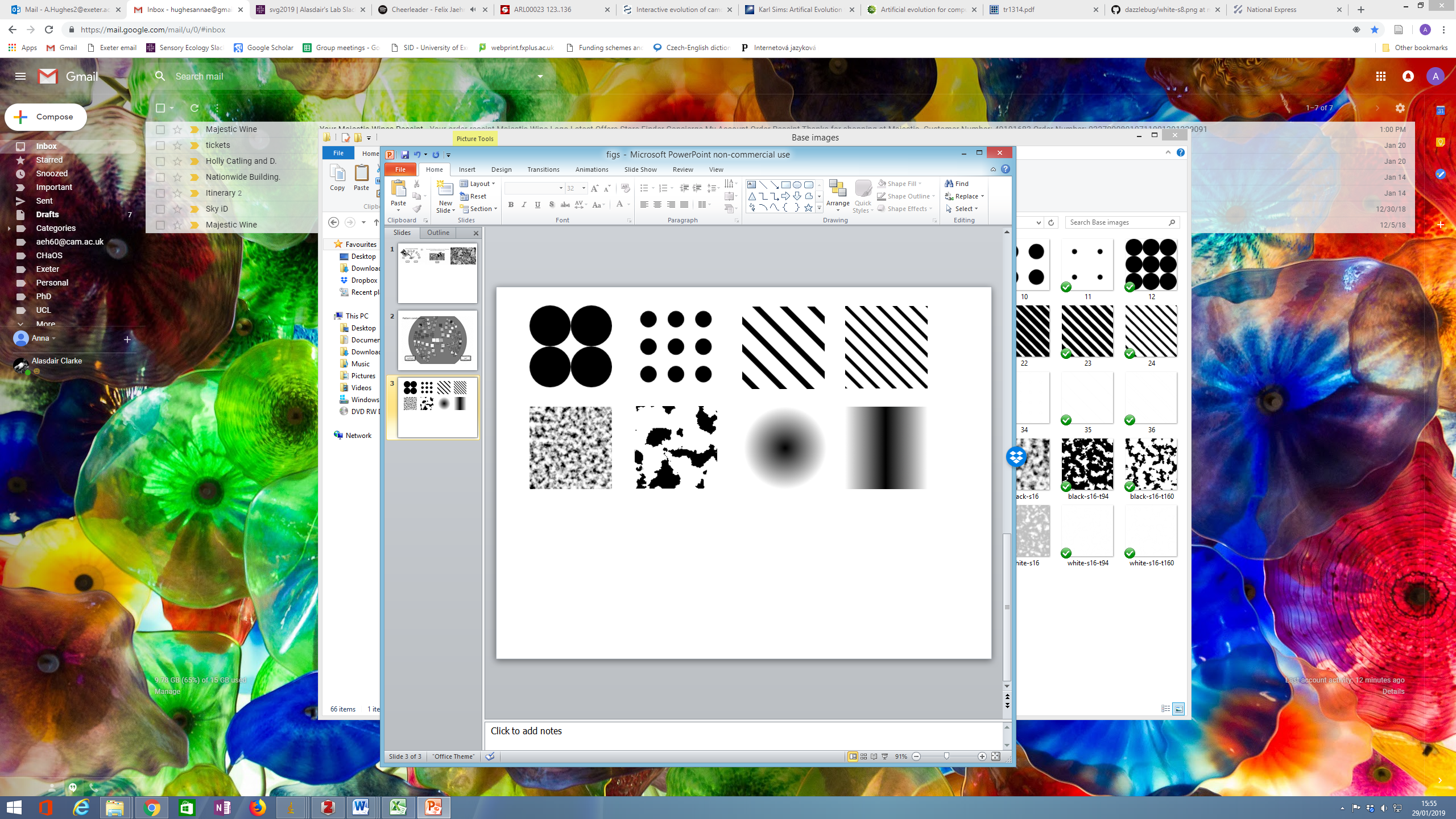


Figure S2: Selection of example base pre-generated images, including spots, stripes, noise and gradients.

An example displayed target is shown in the centre of the screen in Figure S1, and was formed by the top combination node of the tree. The input to this combination node could either be other combination nodes (as seen in this example) or could include a terminal node as well (with 20% probability). The process can be followed backwards until the input to a combination node is two terminal nodes (with randomly chosen parameter inputs), ending that part of the ‘tree’ and forming an outer edge of base images.

A full set of targets for two generations (the medium replicates for generation 1 of the null model) is shown in Figure S3.


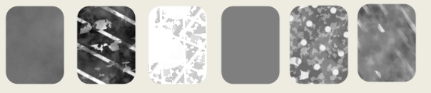

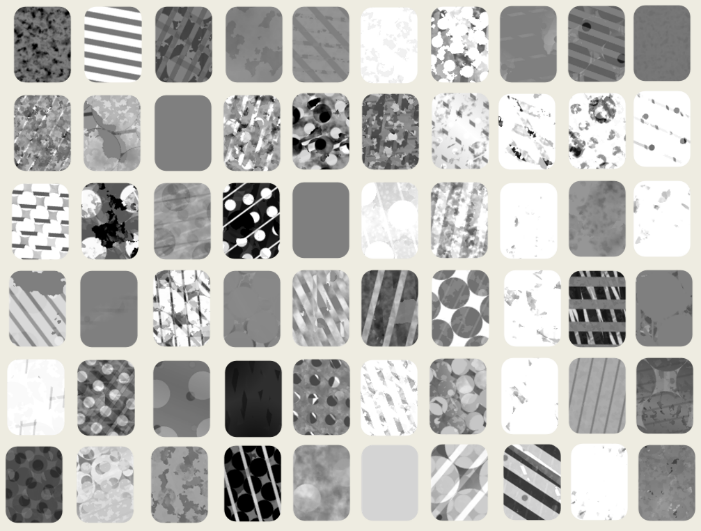

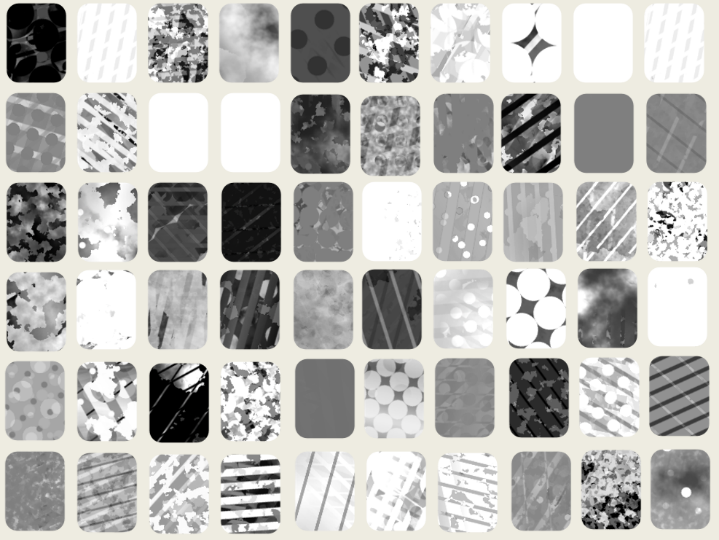

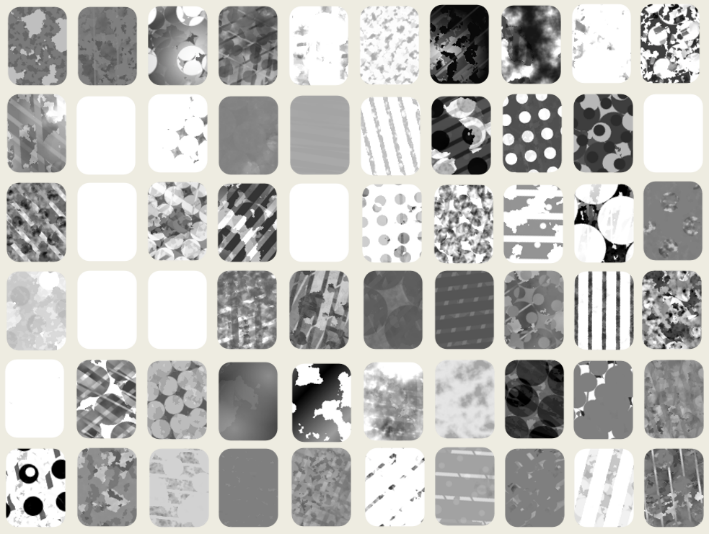

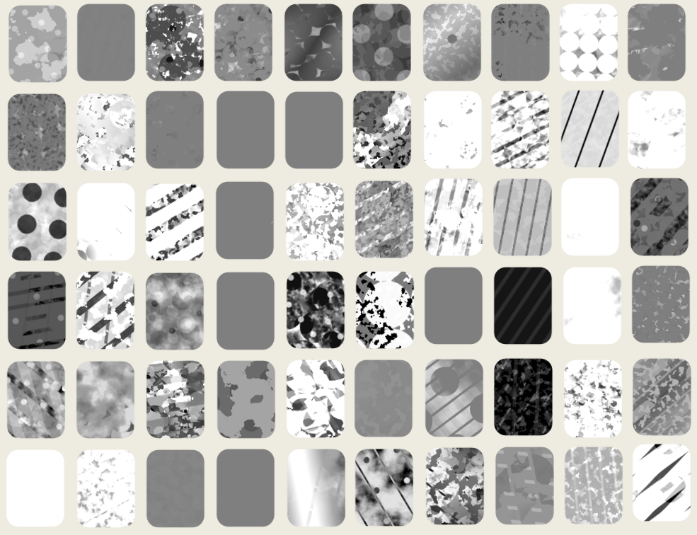


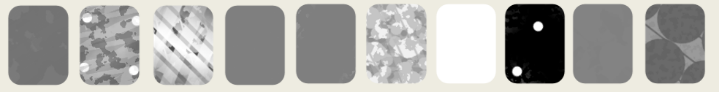


Figure S3: The full set of stimuli for the two replicates of generation 1 of the ‘medium’ population bugs in the null model, showing the range of patterning types that can be generated by the pattern construction process.

**S2. Mutation process**

The mutation process involved either random changes of a parameter variable (e.g. changing the RGB colour, changing the alpha value or changing the pre-generated image) or selecting a random part of the tree (either a combination node or a terminal node), copying it and pasting it onto another random part of the tree. Pruning then occurred if the mutation process increased the depth of the tree to beyond the maximum permitted (6 layers). This process could lead to both increases and decreases in target complexity. The mutation rate was randomly selected for each target, with there being a 0-10% chance of a mutation occurring, but with the probability being weighted towards 0% (i.e. no mutation was most likely, but up to a 10% chance was possible). Mutations did not necessarily have to result in visible differences; it was possible to have ‘genetic’ variation in phenotypically identical targets.

**S3. Quantification and statistical analysis details**

*Determining the best predictors of capture time*

For each measure, we created a model with the log of the average capture time (which can be thought of as the ‘fitness’ of the bug) as the dependent variable. The log transform was used to make this dependent variable Gaussian for use in further analysis. Generation was included as a second order fixed effect to account for non-independence in capture time between generations, and population (fast, medium or slow) was also included as a fixed effect. Replicate ID was included as a random effect. Model Akaike Information Criterion (AIC) values were compared to select the best model and thus determine which measures best predicted capture times (Johnson and Omland 2004), within different categories: for luminance measures, this was the standard deviation of the luminance, a sigma value (spatial frequency of the Gabor filtering method) of 4 for vertical stripes, a sigma value of 2 for horizontal stripes, a sigma value of 2 for diagonal stripes (with both diagonal directions pooled together) and for edge measures, a sigma value of 8 for the right hand edge. In all of these cases, the measure was a highly significant predictor of average fitness (p < 0.001 for all measures). An example of the model structure used is as follows:

lmer(log(Fitness) ~ poly(Generation,2) + Population + scale(SD) + (1|Replicate)

*Modelling changes in fitness across generations and populations in experimental data:*

Comparing a model (via nested model comparison) with the second order effect of generation to one using just the first order effect showed that the more complex model was significantly better (χ^2^ = 13.667, p < 0.001). Therefore, we fit a model with the log of fitness as the dependent variable, and the second order effect of generation and the first order effect of population as fixed factors. Replicate number was included as a random slope.

|  | Estimate | Standard error |
| --- | --- | --- |
| Intercept | 7.729 | 0.02836 |
| Poly(Generation, 2)1 | 5.511 | 0.3996 |
| Poly(Generation,2)2 | -1.352 | 0.3660 |
| Medium population | -0.3771 | 0.002778 |
| Slow population | -0.6224 | 0.002779 |

Table S1: unstandardised effect sizes (regression coefficients) and standard errors for the model assessing changes in fitness across generations and populations in the experimental data.

*Modelling changes in fitness across experimental and control data:*

We fit a similar model as above, but also included a variable indicating whether the data belonged to a null or an experimental population (‘control’). The interaction between generation number and the ‘control’ variable was also included as the key interaction determining whether the increase in fitness was significantly different in the experimental population. Replicate ID was included as a random effect. This model also included only the first 40 generations.

|  | Estimate | Standard error |
| --- | --- | --- |
| Intercept | 7.699 | 0.02588 |
| Poly(Generation, 2)1 | 0.6465 | 0.4315 |
| Poly(Generation,2)2 | -9.645 | 0.4315 |
| Control experiment | -0.02101 | 0.04474 |
| Medium population | -0.3607 | 0.002808 |
| Slow population | -0.5997 | 0.002808 |
| Poly(Generation,2)1 * Control population | -0.4786 | 0.7472 |
| Poly(Generation,2)1 * Control population | 8.136 | 0.7472 |

Table S2: unstandardised effect sizes (regression coefficients) and standard errors for the model of changes in fitness across experimental and control data.


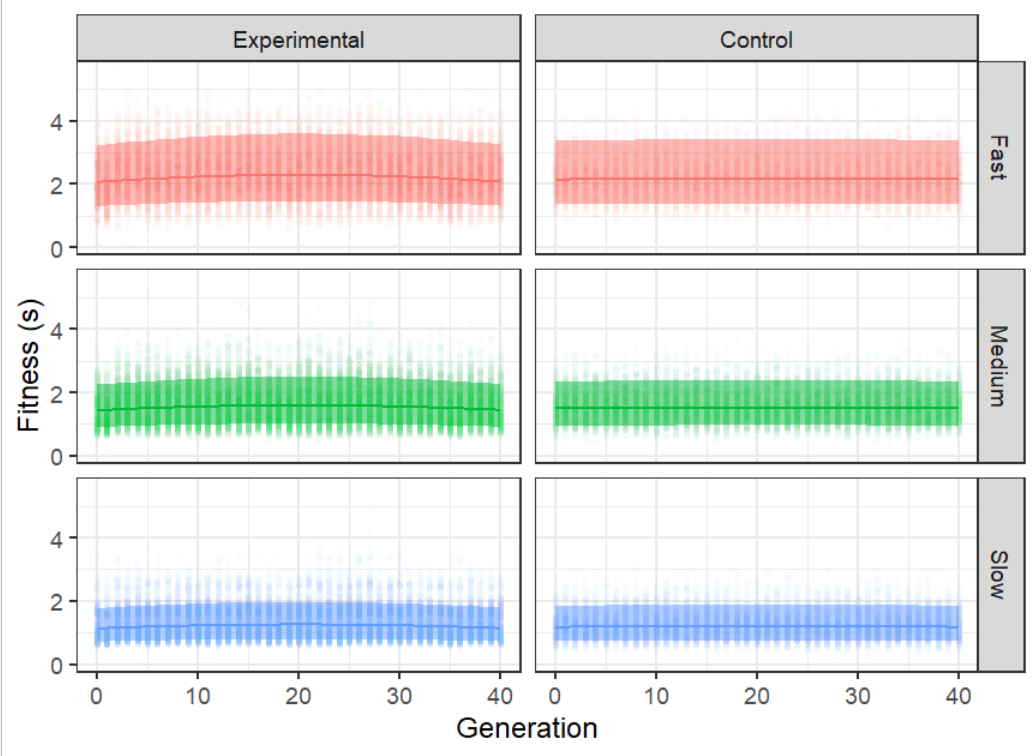


Figure S4: Average fitness (i.e. average time to capture) for both experimental data (left) and control data (right) compared across 40 generations and for the three different speed populations. Experimental data has been collapsed across all 4 replicates. All raw data points are plotted and the lines are fitted as quadratic polynomials on the predicted data from the model. Confidence bands are 80% prediction intervals.

*Analysing how pattern measures changed across experimental and null populations:*

We fit cumulative link models using the ordinal package (version 2019.12-10) (Christensen 2019), with generation as an ordinal dependent variable and the interaction between the pattern measure and the 'control' variable as independent variables. We did not use the patterning measures as dependent variables as these were highly skewed, making it difficult to fit an appropriate model, and we also did not use replicate ID as a random effect as this led to overfitting. The model included the first 40 generations. An example of the model structure used is as follows:

clm(Generation ~ control * scale(SD) )

|  | Estimate | Standard error |
| --- | --- | --- |
| Control population | 0.1848 | 0.0126 |
| Pattern measure (SD) | -0.6792 | 0.0082 |
| Control population * pattern measure | 0.5936 | 0.0122 |

|  | Estimate | Standard error |
| --- | --- | --- |
| Control population | 0.1066 | 0.0123 |
| Pattern measure (vertical stripes) | -0.5078 | 0.0094 |
| Control population * pattern measure | 0.4237 | 0.0125 |

|  | Estimate | Standard error |
| --- | --- | --- |
| Control population | 0.1439 | 0.0124 |
| Pattern measure (horizontal stripes) | -0.6025 | 0.0085 |
| Control population * pattern measure | 0.4543 | 0.0122 |

|  | Estimate | Standard error |
| --- | --- | --- |
| Control population | 0.1750 | 0.0125 |
| Pattern measure (diagonal stripes) | -0.6602 | 0.0090 |
| Control population * pattern measure | 0.5209 | 0.0122 |

|  | Estimate | Standard error |
| --- | --- | --- |
| Control population | 0.1152 | 0.0124 |
| Pattern measure (edge measure) | -0.5469 | 0.0083 |
| Control population * pattern measure | 0.4551 | 0.0123 |

Table S3: unstandardised effect sizes (regression coefficients) and standard errors for the models of pattern change across experimental and null populations. From top: model using the standard deviation of luminance pattern measure, model using the vertical stripe pattern measure, model using the horizontal stripe pattern measure, model using the diagonal stripes pattern measure and model using the edge measure.

*Analysing different selection rates for different speed populations:*

For each combination of population, generation and replicate, we fitted a multiple linear regression between the dependent variable of logged fitness and the five normalised camouflage measures as independent variables. Normalising the camouflage measures ensured that the selection rates for each could be directly compared. We then took the linear regression coefficients for each measure as the linear selection rates. We used these to test for differences in linear selection rates between different speed populations and over evolutionary time (generations). We fitted linear mixed effect models using the linear regression coefficients for each measure as the dependent variable, testing against the second order fixed effect of generation and the fixed effect of population. Replicate ID was included as a random effect, unless this led to overfitting (in which case we ran a simpler linear model). An example of the model structure used was as follows:

lmer(SD_ β ~ poly(Generation,2) + Population + (1|Replicate)

Significance tests for all models were carried out using the ‘Anova’ function from package ‘car’ (version 3.0-10) (Fox and Weisberg 2011) which was used to calculate Type II ANOVAs. Where relevant, post-hoc comparisons were carried out with the 'emmeans' (version 1.5.2-1) package (Lenth 2019).

| Luminance measure | Estimate | Standard error |
| --- | --- | --- |
| Intercept | -0.0831 | 0.0155 |
| Poly(Generation,2)1 | 0.3084 | 0.1986 |
| Poly(Generation,2)2 | 0.6362 | 0.1986 |
| Medium population | 0.0699 | 0.0219 |
| Slow population | 0.0769 | 0.0219 |

| Vertical stripe measure | Estimate | Standard error |
| --- | --- | --- |
| Intercept | -0.0112 | 0.0155 |
| Poly(Generation,2)1 | -0.4568 | 0.1247 |
| Poly(Generation,2)2 | -0.0366 | 0.1247 |
| Medium population | -0.0262 | 0.0138 |
| Slow population | 0.0079 | 0.0138 |

| Horizontal stripe measure | Estimate | Standard error |
| --- | --- | --- |
| Intercept | -0.0378 | 0.0087 |
| Poly(Generation,2)1 | -0.0858 | 0.0980 |
| Poly(Generation,2)2 | 0.0910 | 0.0980 |
| Medium population | 0.0115 | 0.0108 |
| Slow population | 0.0266 | 0.0108 |

| Diagonal stripe measure | Estimate | Standard error |
| --- | --- | --- |
| Intercept | 0.0328 | 0.0165 |
| Poly(Generation,2)1 | 0.1933 | 0.2111 |
| Poly(Generation,2)2 | -0.5216 | 0.2111 |
| Medium population | 0.0010 | 0.0233 |
| Slow population | -0.0273 | 0.0233 |

| Edge measure | Estimate | Standard error |
| --- | --- | --- |
| Intercept | 0.0245 | 0.0187 |
| Poly(Generation,2)1 | -0.4860 | 0.2393 |
| Poly(Generation,2)2 | -0.3005 | 0.2393 |
| Medium population | -0.0630 | 0.0264 |
| Slow population | -0.0333 | 0.0264 |

Table S4: unstandardised effect sizes (regression coefficients) and standard errors for the models analysing selection rate changes for different speed populations. From top: model using the standard deviation of luminance pattern measure, model using the vertical stripe pattern measure, model using the horizontal stripe pattern measure, model using the diagonal stripes pattern measure and model using the edge measure.

**S4. Motion modelling – model set up and statistical analysis details**

*Motion modelling*

The model used was an implementation of a motion model using a two-dimensional array of correlation-type elementary motion detectors (as described in Zanker and Zeil, 2005 and available at <https://github.com/AdamPallus/2dmd>) (Fleishman and Pallus, 2010; How and Zanker, 2014; Pallus et al., 2009). Briefly, the model uses a two-dimensional grid of detectors, in which each detector is compared to another detector a given receptor spacing value away in a vertical or horizontal line. The model is given a series of frames of video in the form of x-y plots of brightness at each pixel. The output from the first detector is delayed with a temporal low pass filter (with a time constant of tau) and multiplied by the instantaneous output from a second detector. For each frame, the operation is performed reciprocally (i.e. in the first operation, the first detector is time delayed, and the second the second detector is delayed) and the difference is subtracted, giving an output magnitude in arbitrary units. Prior to analysis, the input frame is processed with a “Difference of Gaussians” spatial filter to make the average brightness of every frame equal to zero and to enhance the contrast at edges. In the current study, the model was run with the following parameters: a time constant (tau) of 3 frames, size of spacing between receptors equal to 6 pixels, a filter size of 30 pixels and standard deviations of the Gaussians (used for Difference of Gaussians spatial filtering) of 3 and 5 pixels.

*Statistical analysis*

Modelling was carried out using linear models, with the log of fitness being used as the dependent variable, and the coherence (mean resultant), bias (circular mean difference) and the motion energy (average vector length) were used as fixed factors in the model. The interaction between coherence and bias was also included, in line with predictions (How and Zanker 2014). Finally, the data were filtered to include only the points with a circular mean difference of less than 60 degrees. The results were not qualitatively different if these data points were included. To test whether patterning measures could predict the motion energy model output variables, we fit linear models with either the bias or the motion energy as independent variables, and either the standard deviation of the bug luminance or a measure of "stripy-ness"(the energy for vertical filtering angles with a sigma value of 4) as dependent variables.

|  | Estimate | Standard error |
| --- | --- | --- |
| Intercept | 7.9131 | 0.1157 |
| Mean resultant | -3.1374 | 1.3098 |
| Circular mean difference | -0.0092 | 0.0043 |
| Average vector length | -22.880 | 10.2956 |
| Mean resultant * Circular mean difference | 0.1091 | 0.0446 |

Table S5: unstandardised effect sizes (regression coefficients) and standard errors for the statistical model using the motion modelling parameters.
